# Evaluation of Salt Intake Levels and Related Factors in Individuals who Underwent a Specific Health Examination: The LIFE Study

**DOI:** 10.31662/jmaj.2025-0150

**Published:** 2025-09-26

**Authors:** Fumiko Murata, Megumi Maeda, Haruhisa Fukuda

**Affiliations:** 1 Department of Health Care Administration and Management, Graduate School of Medical Sciences, Kyushu University, Fukuoka, Japan

**Keywords:** salt intake, real-world data, health examination

## Abstract

**Introduction:**

High salt intake has been linked to various diseases. However, measuring an individual’s salt intake level may be challenging. This study aimed to identify the characteristics of individuals with high salt intake using a salt intake check sheet.

**Methods:**

This is a cross-sectional study that used data from the Longevity Improvement & Fair Evidence (LIFE) Study. Participants were individuals who underwent a specific medical examination between April 2023 and March 2024 and also completed a salt intake check sheet. Individuals with missing data from the health examinations or the salt intake check sheet were excluded from the study. Individuals taking antihypertensives were also excluded.

Exposure factors were defined as participant sex, age range (40-64 and 65-74 years old), smoking, alcohol consumption, regular exercise, body mass index (BMI; <25 kg/m^2^ or ≥25 kg/m^2^), and the presence of hypertension during specific health examinations (systolic blood pressure ≥140 mmHg or diastolic blood pressure ≥90 mmHg). The salt intake level of participants, based on scores from the salt intake check sheet, was evaluated as the outcome in this study. Using these scores, participants were categorized into two groups: the low salt intake group (0-13 points) and the high salt intake group (>14 points). To evaluate the factors related to salt intake, the salt intake levels (high or low) of participants were set as the response variable, while the exposure factors were set as the explanatory variables. Modified Poisson regression analysis was then conducted.

**Results:**

This study comprised 5,157 participants, with 3,745 in the low salt intake group (72.6%) and 1,412 in the high salt intake group (27.4%). Results from the modified Poisson regression analysis conducted to evaluate the correlation between individual salt intake levels and exposure factors showed statistically significant correlations between the following factors and high salt intake: smoking (relative risk [RR] = 1.35, 95% confidence interval [CI]: 1.21-1.50, p < 0.001); alcohol consumption (RR = 1.19, 95% CI: 1.09-1.30, p < 0.001); and BMI >25 kg/m^2^ (RR = 1.32, 95% CI: 1.19-1.47, p < 0.001). Conversely, participants in the low salt intake group were at lower risk for hypertension compared to those in the high salt intake group; however, the difference was not statistically significant (RR = 0.92, 95% CI: 0.85-1.01, p = 0.08).

**Conclusions:**

The results of this study showed that smoking, alcohol consumption, and having a BMI of ≥25 kg/m^2^ were related to high salt intake.

## Introduction

Excessive salt intake is associated with various lifestyle-related diseases. Previous studies found that a daily salt intake of 10 g results in a blood pressure increase of approximately 6 mmHg over 10 years ^(1)^. Thus, studies found that reducing salt intake is crucial in preventing and managing hypertension ^(2), (3)^. However, the average salt intake in Japan is 9.8 g (10.7 g for males and 9.1 g for females) ^(4)^. These numbers are significantly higher than the daily salt intake of <5 g, as recommended by the World Health Organization ^(5)^, and the daily intake of <6 g, as recommended in the Salt Intake Benchmark for Japanese Households (2025 edition) ^(6)^. To reinforce efforts made towards reducing individual daily salt intake in Japan, a daily salt intake of <7 g in adults has been outlined as a goal in Health Japan 21 (3rd edition). Various policies are being implemented under Health Japan 21, with a central focus on health-related policies ^(7)^.

To achieve the goal of reduced salt intake, appropriate monitoring of an individual’s daily salt intake is indispensable. However, accurately measuring individual salt intake may be challenging. Therefore, alternative methods to identify the degree of salt intake in an individual’s eating habits are necessary. The “My Salt Intake Check Sheet” was developed as a simple tool that allows users to assess their salt intake levels. The salt intake check sheet is a dietary survey method consisting of 13 questions related to dietary habits. The total score is used as a benchmark for individual salt intake levels ^(8), (9)^. The check sheet can be used to identify individuals with high salt intake levels by elucidating the characteristics of such individuals. It also supports the evaluation of effective methods to reduce individual daily salt intake levels. This tool is more practical and feasible in large-scale specific health examinations than objective assessments such as 24-hour urinary sodium excretion. Using this validated and accessible tool aids in identifying individuals with high salt intake and exploring their characteristics in real-world community settings. Moreover, it facilitates public health efforts by providing means to monitor salt intake patterns and evaluate targeted interventions. This study aims to identify the characteristics of individuals with high salt intake using the salt intake check sheet.

## Materials and Methods

### Study design and settings

This study used the data collected during the LIFE Study. The LIFE Study was a multi-site cohort study conducted by Kyushu University. Data were collected on individuals who underwent a specific health examination for research purposes ^(10)^. The specific health examination was conducted with the aim of preventing lifestyle-related diseases. The target population was individuals under the National Health Insurance. The examination focused on metabolic syndromes.

This study received assistance from four municipalities that participated in the LIFE Study. The salt intake check sheet was administered to individuals who underwent a specific healthcare examination during group examinations conducted by these municipalities. The group-specific medical checkups were conducted at municipal health centers as part of the specific medical examinations. Individuals who underwent the specific medical examination were administered a copy of the salt intake check sheet alongside the questionnaire that was distributed prior to the specific medical examinations. The salt intake check sheet was collected during the group-specific medical examination. The participants in this study were individuals who underwent specific medical examinations during group-specific medical examinations from April 2023 to March 2024 and responded to the salt intake check sheet. Individuals who only underwent specific medical examinations or only responded to the salt intake check sheet were excluded from this study. In cases where deficits were found in the data from the health examinations or the salt intake check sheets, affected individuals were also excluded. Similarly, those who answered “yes” to the use of antihypertensives in the specific medical examination questionnaire were excluded, as antihypertensive medication use was considered a proxy for prior hypertension diagnosis and possible lifestyle modifications such as reduced salt intake following medical or nutritional advice.

### Exposure

The following were exposure factors in this study: age range, smoking status, alcohol consumption, regular exercise habits, body mass index (BMI) status, and the presence of hypertension at the time of specific medical examinations. The age groups for participants in this study were 40−64 years and 65−74 years. According to the guidelines established by the Ministry of Health, Labor and Welfare in Japan, which define individuals with > BMI 25 kg/m^2^ as targets for health guidance, this study also classified participants into two groups: BMI <25 kg/m^2^ and ≥25 kg/m^2(11)^. Habits related to smoking, alcohol consumption, and regular exercise were identified using the questionnaire administered during health examinations. Smoking status was based on a questionnaire item asking whether the respondent currently smokes habitually. Regular physical activity was defined as engaging in (1) light sweating exercise for ≥30 minutes at least twice a week for over a year, or (2) walking or equivalent physical activity for ≥1 hour daily. Alcohol consumption was defined as “yes” for individuals who reported drinking alcoholic beverages such as sake, shochu, beer, or western liquors daily or occasionally, and “no” for those who reported rarely drinking. In addition, the variable “presence of hypertension” was defined based on systolic and diastolic blood pressure readings obtained during specific health examinations. According to the guidelines of the Ministry of Health, Labor and Welfare in Japan, blood pressure should be measured twice, with the average representing the health checkup results; however, a single measurement may be used if the situation requires it ^(11)^. High blood pressure was inferred in individuals with either a systolic blood pressure of ≥140 mmHg or a diastolic blood pressure of ≥90 mmHg.

### Outcome

Data obtained by scoring the responses of participants to the salt intake check sheet were used as the outcome in this study. The salt intake check sheet is a self-administered questionnaire comprising 13 questions on eating habits ^(9)^ (Supplemental Table). The respondents were asked to rate their eating habits in terms of frequency and amount on a four-point scale. The score for responses to each question ranged from 0 to 3. These scores were evaluated with the highest possible total score being 35. The questions were categorized as follows: seven items were used to evaluate the intake of salty meals such as miso soup, pickles, and noodles; four items were used to assess the use of salty sauces (e.g., soy sauce), frequency of eating out, and use of home-meal replacements; two items were used to address the seasoning intensity and portion size of home-cooked meals. Participants’ scores were categorized as follows: 0-8 (low levels of salt intake), 9-13 (average levels of salt intake), 14-19 (high levels of salt intake), and ≥20 (very high levels of salt intake). In this study, participants who scored between 0-8 and 9-13 were included in the low salt intake group, while those who scored between 14-19 or >20 were included in the high salt intake group.

### Statistical analysis

The descriptive statistics for categorical variables were presented in percentages (%). The differences in distribution for each variable in both participant groups were presented using the χ^2^ test. In addition, to evaluate exposure factors influencing individual salt intake levels, these levels (high or low) were set as the object variable, and explanatory variables were treated as exposures. Modified Poisson regression analysis was conducted. Statistical analyses were performed using Stata version 17.0 (Stata Corp, College Station, TX, USA), and significance was set at p < 0.05. The study was approved by the Kyushu University Institutional Review Board for Clinical Research (number 22114-09). Given that this study was a retrospective study involving anonymized data, the requirement for informed consent was waived, and an opt-out approach was employed to ensure the protection of participants’ rights.

## Results

**Figure 1.**
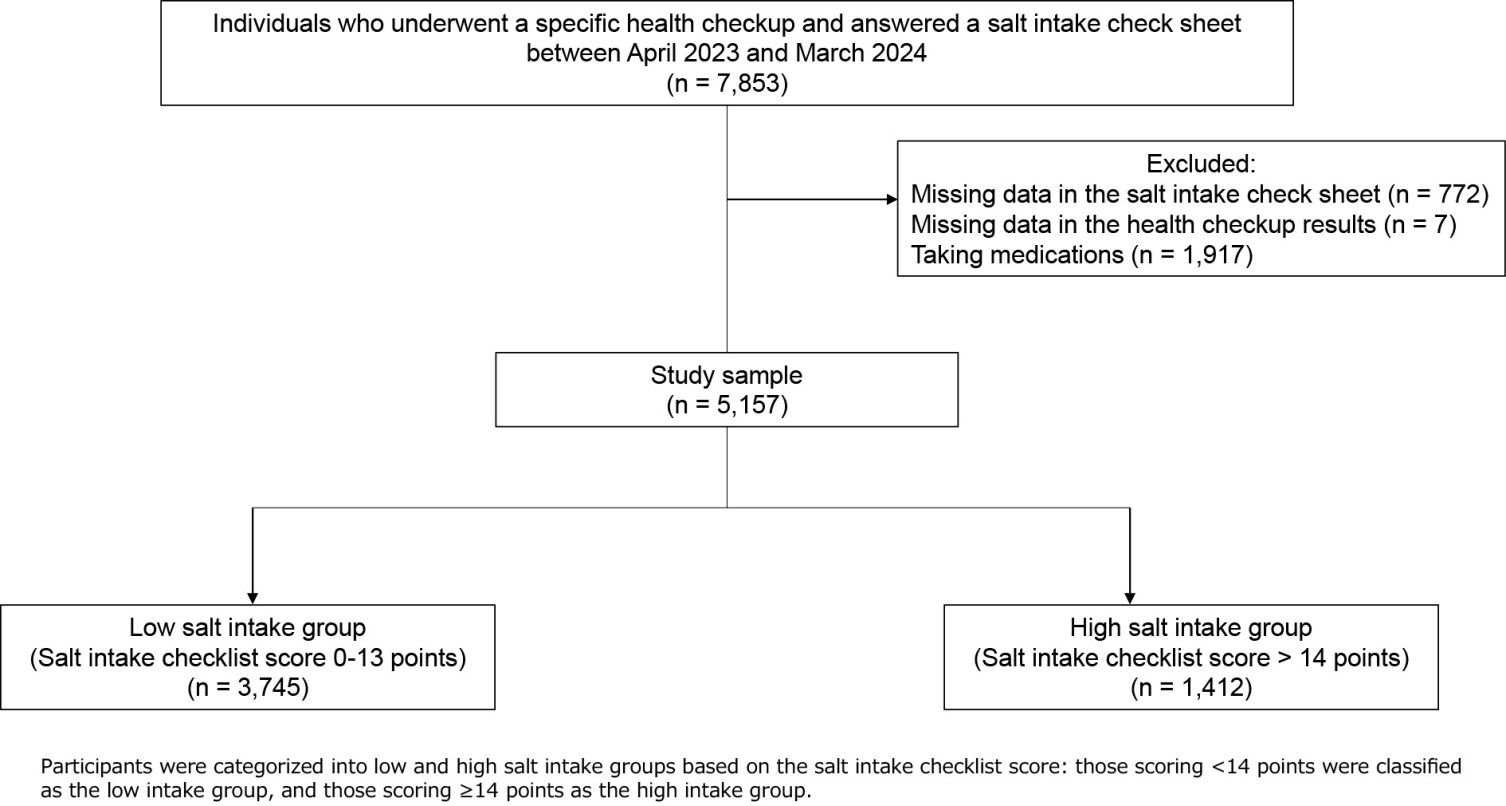
Flowchart of study participant selection.

Overall, 5,157 individuals participated in this study (Figure 1). Participant’s characteristics are displayed in Table 1. The low salt intake group comprised 3,745 individuals (72.6%), while the high salt intake group comprised 1,412 individuals (27.4%). Two thousand five hundred three individuals in the low salt intake group (66.8%) and 649 in the high intake group (46.0%) were females. Regarding age group, 2,309 individuals (61.7%) in the low salt intake group and 781 individuals (55.3%) in the high salt intake group were aged 65-74. The proportion of individuals with a BMI of >25 kg/m^2^ was 503 (13.4%) in the low salt intake group and 313 (22.2%) in the high salt intake group. A total of 721 individuals in the low salt intake group (19.3%) and 288 in the high intake group (20.4%) had hypertension.

**Table 1.** Participant Characteristics.

XXX	Low salt intake group (n = 3,745)	High salt intake group (n = 1,412)	*p*-Value
Female	2,503 (66.8)	649 (46.0)	<0.001
Age group			
40-64 years	1,436 (38.3)	631 (44.7)	<0.001
65-74 years	2,309 (61.7)	781 (55.3)	
Smokes	349 (9.3)	268 (19.0)	<0.001
Consumes alcohol	1,788 (47.7)	823 (58.3)	<0.001
Exercises regularly	2,508 (67.0)	963 (68.2)
BMI			
<25 kg/m^2^	3,242 (86.6)	1,099 (77.8)	<0.001
≥25 kg/m^2^	503 (13.4)	313 (22.2)	
Hypertension	721 (19.3)	288 (20.4)	0.356
Systolic BP (mmHg), mean [SD]	123.4 [18.9]	124.4 [18.2]	0.105
Diastolic BP (mmHg), mean [SD]	75.5 [10.6]	76.7 [11.0]	<0.001

Values are presented as number (%) unless otherwise indicated. BMI: body mass index; BP: blood pressure; SD: standard deviation.

The result of the modified Poisson regression analysis conducted to evaluate the correlation between individual salt intake levels and exposure factors is shown in Table 2. To compare the high salt intake group with the low salt intake group, statistically significant correlations were found between the following factors and high salt intake: smoking (relative risk [RR] = 1.35, 95% confidence interval [CI]: 1.21-1.50, p < 0.001), alcohol consumption (RR = 1.19, 95% CI: 1.09-1.30, p < 0.001), and BMI ≥25 kg/m^2^ (RR = 1.32, 95% CI: 1.19-1.47, p < 0.001). Conversely, the low salt intake group showed a lower risk for hypertension compared to the high salt intake group. However, the difference was not statistically significant (RR = 0.92, 95% CI: 0.85-1.01, p = 0.08).

**Table 2.** Correlation Between Individual Salt Intake Levels and Exposure Factors.

XXX	RR (95% CI)	*p*-Value
Sex		
Female	0.62 (0.56-0.68)	<0.001
Age group		
40-64 years	Reference	
65-74 years	0.88 (0.80-0.96)	0.004
Smokes	1.35 (1.21-1.50)	<0.001
Consumes alcohol	1.19 (1.09-1.30)	<0.001
Exercises regularly	1.06 (0.97-1.17)	0.209
BMI		
<25 kg/m^2^	Reference	
≥25 kg/m^2^	1.32 (1.19-1.47)	<0.001
Hypertension	0.97 (0.87-1.08)	0.531

The modified Poisson regression was adjusted for the following: participant sex, age group, smoking, alcohol consumption, regular exercise, BMI status, hypertension. BMI: body mass index; CI: confidence interval; RR: relative risk.

## Discussion

This study used data obtained from the ‘salt intake check sheet’ and a specific medical examination to identify risk factors related to high salt intake. Results showed that smoking, alcohol consumption, and a BMI of ≥25 kg/m^2^ were correlated with high salt intake. Conversely, increased salt intake did not correlate with hypertension.

Reportedly, smokers prefer salty foods; therefore, they tend to consume higher amounts of salt ^(12)^. Furthermore, smokers have been identified to consume more alcohol than non-smokers ^(13)^. Studies have evidenced that individuals who smoke and consume alcohol are at a higher risk for excessive salt intake than individuals who do not smoke or consume alcohol ^(14)^. Individual BMI scores have also been shown to increase as salt intake increases ^(15)^. The current findings align with previous studies, showing that high salt intake levels may be correlated with smoking, alcohol consumption, or a BMI of ≥25 kg/m^2^.

Conversely, previous studies have shown a correlation between high salt intake and hypertension ^(2), (3)^; however, this correlation was not found in this study. There are two possible explanations for this discrepancy. Firstly, it is believed that there is a time difference before the onset of hypertension depending on individual salt intake levels. However, salt intake levels and the presence of hypertension were assessed simultaneously during this study. Hence, individuals with high salt intake levels did not display symptoms of hypertension. However, these individuals may exhibit symptoms of hypertension a few years later. This time difference was not evaluated in the study. Secondly, the salt intake check sheet was administered only once, limiting its usefulness in evaluations conducted over an extended period. Individuals who reduce their salt intake while monitoring their blood pressure may still be hypertensive. This bias is not caused by using the salt intake check sheet, as it may still occur even if the actual conditions surrounding individual salt intake levels have been accurately evaluated. Thus, medium- or long-term tracking of hypertension symptoms and continuous evaluation of salt intake levels are necessary to assess the correlation between salt intake levels and high blood pressure. Moving forward, it will be necessary to gather more detailed data spanning a longer period.

This study had a few limitations. First, the data used in this study were gathered only from four municipalities; hence, the study results cannot be generalized to other regions. Second, participants in this study were limited to individuals insured under the national health insurance and who underwent specific medical examination. Therefore, the results may not be applied to other population groups. Generally, the income level of individuals insured under the national health insurance may be low. Furthermore, individuals who have undergone specific medical examinations have higher rates of hospital visits compared to those who have not. Additionally, participants in the former group are highly health conscious, considering that individual medical costs are low ^(16)^. Third, information related to stress, which influences hypertension ^(17)^, and hereditary factors ^(18)^ was not included in this study. These data were not available to the local municipalities; therefore, it may be challenging to conduct further analysis. Fourth, salt intake levels were measured using a questionnaire; however, it is difficult to accurately measure actual individual salt intake levels. Moreover, these potential biases may still occur even if individual salt intake levels have been measured accurately.

### Conclusions

The results of this study showed that smoking, alcohol consumption, and having a BMI of ≥25 kg/m^2^ were related to high salt intake.

## Article Information

### Acknowledgments

The authors are grateful to the staff of the four participating municipalities for their cooperation in this study.

### Author Contributions

Designed the study and collected the data: Fumiko Murata, Megumi Maeda, Haruhisa Fukuda. Performed the analysis: Fumiko Murata. Interpreted the results: Fumiko Murata, Megumi Maeda, Haruhisa Fukuda. Drafted the original manuscript: Fumiko Murata. Reviewed and edited the manuscript: Fumiko Murata, Megumi Maeda, Haruhisa Fukuda. Supervised the study: Haruhisa Fukuda. Read the manuscript and approved its submission for publication: Fumiko Murata, Megumi Maeda, Haruhisa Fukuda.

### Conflicts of Interest

None

### Data Sharing

Data cannot be shared for privacy or ethical reasons.

## Supplement

Supplementary Material
