# Supplementary material for: Evaluation of Salt Intake Levels and Related Factors in Individuals who Underwent a Specific Health Examination: The LIFE Study
Source: JMA J. 2025 Sep 26;8(4):1214–9. doi: 10.31662/jmaj.2025-0150 (PMC12598292; doi:10.31662/jmaj.2025-0150)
Supplement: Supplementary Material [file 2433-3298-8-4-1214-s001.pdf]

Supplemental Table Salt check sheet. Your salt check sheet, circle what fits you and write the total score at the end

		3 points	2 points	1 point	0
Frequency of eating these foods	miso (fermented soybean paste) soup, soup, etc.	more than 2 bowls a day	about 1 bowl a day	two-three bowls a week	hardly eat
	pickles, pickled plums, etc.	more than twice a day	about once a day	two-three times a week	hardly eat
	fish-paste products such as *chikuwa* (tubular fish sausage) and *kamaboko* (steamed fish paste)		eat frequently	two-three times a week	hardly eat
	horse mackerel cut open lengthwise and dried,dried fish seasoned with mirin (sweetened alcohol for use in cooking), salted salmon, etc.		eat frequently	two-three times a week	hardly eat
	ham or sausage		eat frequently	two-three times a week	hardly eat
	noodles such as *udon* (Japanese wheat noodles) and *ramen* (Japanese-style Chinese noodles)	almost every day	2 or 3bowls a week	less than once a week	don't eat
	senbei (Japanese crackers), okaki(thinly-cut and dried rice cakes),potato chips,etc.		frequently	two-three times a week	hardly eat
How frequent do you season with soy sauce or other sauces?		season frequently (almost every meal)	once a day	season sometimes	don't season
How much udon , ramen , or other soups do you consume?		entire bowl	about half	some	little
Do you eat out or have convenience-store-bought bento (lunch plate) for lunch?		almost every day	about 3 times a week	about once a week	no
Do you eat out or have ready-made side dishes for dinner?		almost every day	about 3 times a week	about once a week	no
How salty are your home-made dishes compared with those you eat out?		heavy	same		light
Do you think you eat a lot?		more than others		same as others	less than others

total score of the items you circled	3 points×__	2 points×__	1 point×__	0 point×__
subtotal	__points	__points	__points	__points
total points	___points			

check below☒	total points	evaluation
☐	0-8	You are not taking excess salt. Maintain this diet for salt restriction.
☐	9-13	Your salt intake is average. Start a little stricter salt restriction.
☐	14-19	You have excessive salt intake. You need to lower your salt intake by changing your diet salt content and eating behavior.
☐	20-	You have too much salt intake. You need to totally change your diet salt content and eating behavior.
